# Acute Effects of Work Rest Interval Duration of 3 HIIT Protocols on Cycling Power in Trained Young Adults

**DOI:** 10.3390/ijerph18084225

**Published:** 2021-04-16

**Authors:** José Manuel García-De Frutos, Fco. Javier Orquín-Castrillón, Pablo Jorge Marcos-Pardo, Jacobo Á. Rubio-Arias, Alejandro Martínez-Rodríguez

**Affiliations:** 1 Physical Activity and Sport Sciences Department, Faculty of Sport, Catholic University San Antonio of Murcia, 30107 Murcia, Spain; defrutos60@gmail.com (J.M.G.-D.F.); forquin@ucam.edu (F.J.O.-C.); 2 Department of Education, Faculty of Education Sciences, University of Almería, 04120 Almería, Spain; pjmarcos@ual.es (P.J.M.-P.); jararias@ual.es (J.Á.R.-A.); 3 SPORT Research Group (CTS-1024), CERNEP Research Center, University of Almería, 04120 Almería, Spain; 4 Department of Analytical Chemistry, Nutrition and Food Sciences, Faculty of Science, Universidad de Alicante, 03690 Alicante, Spain; 5 Alicante Institute for Health and Biomedical Research (ISABIAL Foundation), 03010 Alicante, Spain

**Keywords:** power loss, blood lactate concentration, youth, athletic performance

## Abstract

High-Intensity Interval Training (HIIT) is described as a succession of short duration and maximum or near-maximum intensity efforts, alternated by recovery periods during which exercise continues at a lower intensity (active recovery) or is interrupted (passive recovery). Our objective was to evaluate the acute responses of three HIIT protocols of different work/rest interval times over the total time of the session, with self-selectable load and up to exhaustion, “all out”.The sample was composed of 22 male participants (*n* = 22) between 19 and 24 years old. The HIIT protocol consisted of one of the three HIIT protocols, of 30, 60 and 90 s density ratio 1:1 and with passive rest, with a total exercise duration of 10 min. The test was performed in a cycloergometer set in workload mode independent of the pedaling frequency. The comparison of the three HIIT protocols shows that the duration of the work/rest intervals, starting from 30 s of work, in the cycloergometer, there are no significant differences in the levels of lactate concentration in the blood, nor in the heart rate, since a similar amount is obtained in the three protocols. The percentage of maximum power developed reached in each HIIT protocol is related to the duration of the working intervals.

## 1. Introduction

High-intensity interval training (HIIT) is a training method currently considered one of the most effective in improving cardiorespiratory and metabolic function [1]. HIIT can be defined as repeated bouts of high intensity exercise, from a maximum lactate steady state (MLSS) respiratory compensation point (CPR) to a total supramaximal exercise intensity, interspersed with periods of recovery from low or moderate intensity exercise (active recovery) or complete rest [2]. Due to these recovery phases, peak workloads can be sustained for a longer cumulative period than in a period of continuous exercise [3].

In addition, HIIT is considered the most effective training modality to improve cardiorespiratory and metabolic functioning and, in turn, endurance performance [1] since this exercise modality allows individuals to perform a greater volume of exercises at high intensity, than do continuous exercises [4,5]. With exercises at these intensities, the oxygen delivery and utilization systems are under maximal stress, which may provide the most effective stimulus for increasing maximum oxygen uptake (VO2max) [1,6].

According to Seiler [7] the intensity of exercise can be divided into three zones: exercise intensity zone 1, which corresponds to an intensity with minimal accumulation of lactate in the blood, zone 2, which corresponds to an intensity with accumulation of lactate in the blood (below the lactate threshold (LT) or ventilatory threshold (VT)), but with a rate in the exercise that remains sustainable with effort (below maximal lactate steady state (MLSS)/ onset of blood lactate accumulation (OBLA)), and zone 3, which corresponds to an intensity that cannot be maintained for more than 5 min, and therefore we define it as HIIT. However, the goal of HIIT is defined as increasing the training time close to VO2max, thus producing a stronger stimulus for cardiovascular health and muscle adaptations [1].

The prescription of a HIIT consists of the manipulation of up to nine variables including intensity and duration of the work interval, intensity of the recovery interval, and duration, type of exercise, number of intervals, time of series, recovery between series duration and intensity. The manipulation of any of them can affect the acute physiological responses of the subject [1]. Currently, the scientific community shows a diversity of HIIT methods applied in different studies, with different types of samples and protocols which suggest that the acute physiological mechanisms during HIIT are not clear, or that all their possibilities are not taken into account [8]. The degree of these acute physiological responses is strongly influenced by the specific prescription of single exercise, components such as intensity and duration of peak workload and recovery phases [7], although isolated manipulation of a single variable during exercise can have a direct impact on acute metabolic, cardiopulmonary and neuromuscular responses [7] it is the acute physiological response that largely determines the particular muscular and systemic training adaptations [9,10]

Consequently, a consistent prescription model is needed to regulate and predict the acute physiological responses of HIIT [11]. Interval training protocols can be divided into two categories, based on intensity-based exercise [12]: HIIT, performed at intensities between 80% and 100% of maximum work capacity interspersed with periods of low active recovery intensity of 1 to 4 min, and SIT, characterized by total intervals at intensities of 100% of maximum work capacity interspersed with 2–4 min of low intensity active/passive recovery.

Despite the growing number of research studies employing protocols varying from submaximal intensity (HIIT) to supramaximal intensity (SIT), little is known about physiological and psychological responses. Understanding how HIIT and SIT protocols that vary in work/rest ratio and intensity differentially influence psychophysiological responses is essential to effectively prescribe HIIT and SIT for both health/fitness and performance training [13].

However, methodological issues such as small sample size, short intervention period, and/or matching training regimen to total energy expenditure make it difficult to compare results [14].

Therefore, it is imperative for fitness professionals that physiological responses to various protocols and populations are characterized so that HIIT can be safely prescribed [15]. Even less is known about the acute response in different “all out” HIIT protocols participants must require to self-select their exercise intensity in response to “interval and maximal effort session” [16,17,18] protocols of the same duration, but with different working time, specifically in young active men.

The purpose of this study was to compare the pre–post acute response of 3 protocols of different work time duration, but with the same total work time, in young men active on bicycle ergometers. This objective was to test the hypothesis that the longer the work duration, the lower the acute response in testing.

## 2. Materials and Methods

### 2.1. Study Design

**Figure 1:**
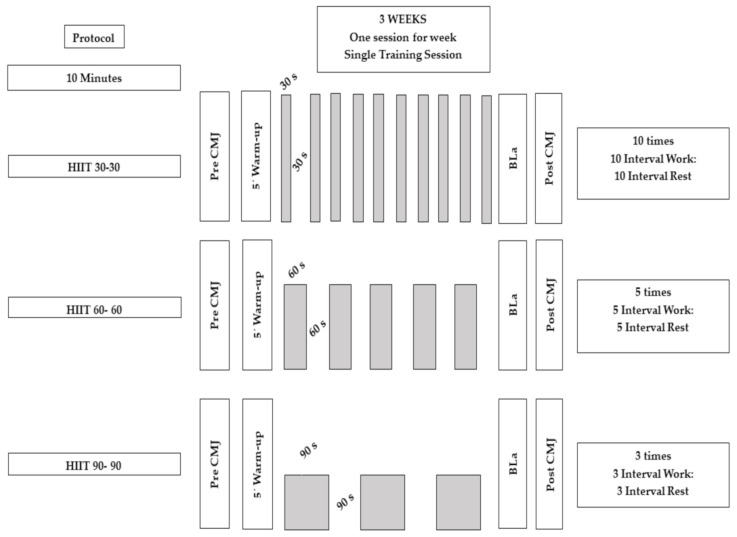
Scheme of the training sessions. BLa = Lactate; CMJ = Counter movement jump.

A cross-over design study was conducted with an experimental group where participants completed 3 counterbalanced HIIT sessions at 3 different times, developed one per week (see Figure 1). The study followed the ethical research standards as established in the Declaration of Helsinki and was approved by the local ethical committee (San Antonio Catholic University of Murcia, Spain) with the code: C8061811.

### 2.2. Participants

Twenty-two young men (mean age: 21 ± 2.0 years; body weight: 70.6 ± 11.1 kg; body height: 174.2 ± 9.9 cm; body mass index: 24.7 ± 1.7 kg/m^2^; HIIT training experience: 1 year, training: 2 h/week) had previous experience with high-intensity training. All-out concept was introduced as maximum effort developed and maintained for the time of work. The inclusion criteria in the study were physically active experts in the HIIT training method. The inclusion criteria in the study were: (1) being between 18 and 25 years old, (2) being active according to the American College of Sports Medicine (ACSM) definition [19], (3) having experience of more than 6 months of training in HIIT method, and (4) training systematically with the HIIT method. The exclusion criteria were: (1) ingesting any type of pharmacological treatment that may influence the performance (improvement or decline) of the subject, (2) performing other physical activities with overload that may influence the results of the study during participation, and (3) not respecting the training guidelines dictated in the study. All participants gave their informed written consent to participate in the research. 

Participants were instructed to avoid exercising for a minimum of 24 h and consuming alcoholic beverages for a minimum of 48 h prior to testing. An initial information session was held during which were detailed the study design (including the study objectives) and the dates of the pre- and post-intervention evaluations. During this session, the testing protocol and the intervention plan were detailed to the participants. The intervention program took place once a week for three weeks in a row. 

### 2.3. Training Intervention

Training interventions (every single session) were supervised by professional sport scientists. The experimental group (EG) had to carry out 3 supervised training sessions. Such sessions took place on the same day of the week once a week and chronologically followed an established order. Each session lasted about 40 min. The same warm-up was performed in all sessions, consisting of 5 min of continuous pedaling on the cycle ergometer, at 100 W, with 1 min of rest [7].

The 3 protocols of the intervention consisted of a HIIT session of 30, 60, and 90 s, respectively. The 3 protocols held the same 1: 1 density, passive rest, and total duration of 10 min. An electronically controlled cycle ergometer (Technogym Bike Med Technogym SpA, Cesena, Italy) was employed, with a workload mode independent of pedaling frequency [14]. In all protocols, subjects had to self-select their exercise intensity in response to a prescription of “effort maximum interval and session” [14]. Participants were instructed to remain seated on the cycle ergometer during the test. 

In total, 10, 5, or 3 high intensity intervals were performed according to the protocol of 30 s, 60 s, or 90 s, respectively, analyzing the maximum power (Pmax), average power (Pmean), and power loss (Ploss), all measured in watios (W) between intervals, taking the value of said power every 5 s during the HIIT work intervals. All 22 participants completed the 3 sessions without incidents. 

### 2.4. Anthropometric Measurements

Anthropometric measurements were performed in fasting conditions. Height (cm) and body mass (kg) were assessed using a digital balance and portable stadiometer. Body mass index (BMI) was calculated using the equation “body mass (kg)/height (cm)^2^”.

### 2.5. Physical Measurements Control

Heart rate (HR) and blood lactate concentration (BLa) levels were used as indicators of the intensity of the test. HR was measured continuously by analyzing the mean in each work interval by telemetry (Polar H7, Beth Page, NY, USA) in all sessions, during the test time. After a 1-min rest, capillary BLa was measured with the enzymatic amperometric method using the Lactate Scout system (RedMed, Warsaw, Poland). The test range for the method was 0.5–25 mmol/L. The tip of the index finger was washed with a wet paper towel and dried, and the lactate concentration was measured using the second drop of blood, since the first drop was removed.

### 2.6. Physical Measurements Experimental 

In order to obtain indicators of the subject’s performance in each of the HIIT protocols, the explosive force of the lower body was measured, by comparing the maximum height reached in the performance of a Counter Movements Jump (CMJ) test [20], pre and post completion of each HIIT protocol, using the MuscleLab force platform (Ergotest Technology AS, Porsgrunn, Norway) and data collection of the different results of the power achieved on the cycle ergometer during each interval of each HIIT protocol. Pmax, Pmean, and Ploss were all measured in watios (W).

### 2.7. Statistical Analysis

For statistical analysis, the IBM SPSS Statistics for Windows program in version 25.0 (Armonk, NY: IBM Corp.) was used. Descriptive statistics were reported as mean ± standard deviation. Preliminary analyses included checking assumptions such as normality, homogeneity of variances. No violations of the normality of the data were observed in the Kolmogorov–Smirnov test, which led to the use of parametric statistics. The homogeneity of the variances was carried out using Levene’s test. Likewise, all variables were analyzed according to the two-way ANOVA test (time: before or after; and protocol) to examine whether there were statistically significant differences in these types of variables between the protocols.

However, in relation to the development of power in each of the protocols, independently, a comparison was carried out between the different intervals that made up each of the protocols by means of a repeated measures ANOVA. For all variables, the level of statistical significance was established at *p* ≤ 0.05, with a 95% confidence interval for differences. The effect size (ES) was calculated using the partial eta-square statistic (ηp2), considered greater than 0.8, between 0.8 and 0.5, between 0.5 and 0.2, and less than 0.2 were considered large, moderate, small, and trivial, respectively [21].

## 3. Results

In the results obtained in the physical control measurements, in blood lactate concentration level, no significant differentiation was observed when the lactic acid concentration was compared at the three protocols (F = 1000; *p* = 0.337; ηp2 = 0.031). On the other hand, in the variable of the mean heart rate, there were significant differences (F = 10.344; *p* = 0.000; ηp2 = 0.247) between the 30 s vs. 90 s protocols (*p* < 0.001; 95% CI = (4.5286–14.6533); ηp2 = 0.247). Table 1 shows the physiological control parameters chosen at the baseline for the 3 different training protocols, whereas Table 2 presents the experimental physiological parameters chosen at the baseline. 

The results found for the experimental physical measurements show no significant differences in the increase in height in the CMJ when the three HIIT protocols were compared (F = 0.385; *p* = 0.682; ηp = 0.012). For increased power in the CMJ, no significant differences were found when the three protocols were compared (F = 0.185; *p* = 0.832; ηp2 = 0.006).

**Figure 2:**
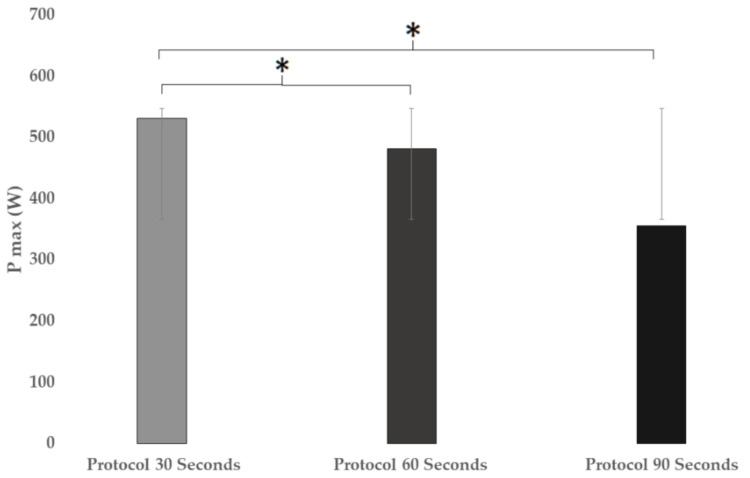
Comparison of maximum power developed in different High-Intensity Interval Training (HIIT) protocols. * means significant difference.

Regarding the power developed during the 3 protocols (see Figure 2), significant differences were found in the maximum power (F = 9.237; *p* < 0.001; ηp2 = 0.227). In the comparison between the protocols, there were significant differences between the 30 s vs. 90 s groups (*p* < 0.001) of the multiple comparison (*p* < 0.001; ηp2 = 0.227; 95% CI = (74.7252–277.5475)). There were also significant differences between the 90 s vs. 60 s protocols (*p* = 0.011; ηp2 = 0.227; 95% CI = (−227.7293–−24.9071).

**Figure 3:**
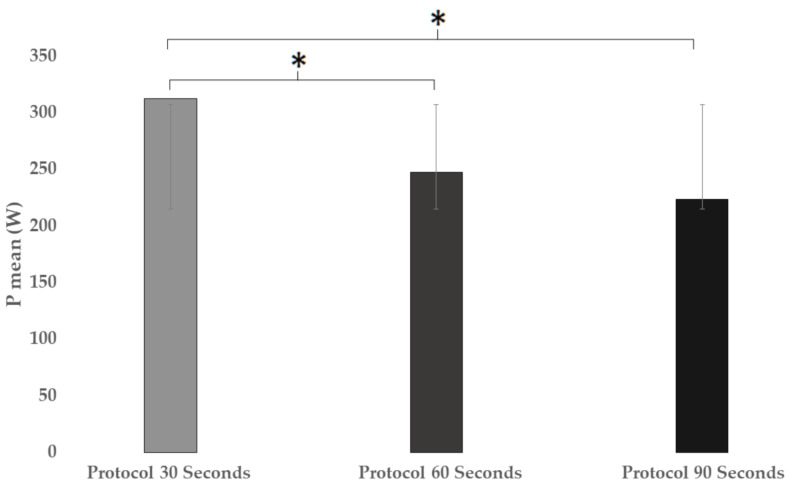
Average power comparison developed in different HIIT protocols. * means significant difference.

In the mean power (Figure 3), significant differences were found when the three protocols were compared (F = 14.926; *p* < 0.001; ηp2 = 0.322). In the comparison between the protocols, significant differences were found between groups 30 and 60 s (*p* = 0.001) of the multiple comparison (*p* < 0.001; ηp2 = 0.322; 95% CI = (24.7917–105.9356)) and between protocols 30 and 90 s (*p* < 0.001) of the multiple comparison (*p* < 0.001; ηp2 = 0.322; CI95% = (48.6098–129.7538)).

**Figure 4:**
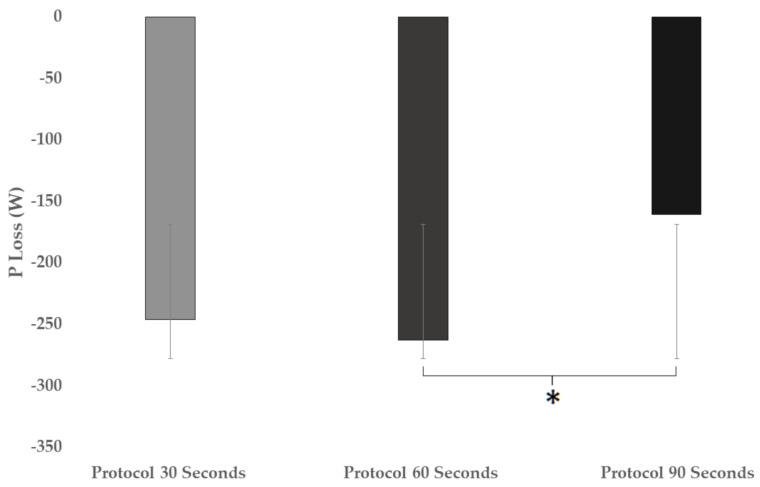
Loss power comparison developed in different HIIT protocols. * means significant difference.

In the variable of lost power (Figure 4), significant differences were found when the three protocols were compared (F = 3.217; *p* = 0.047; ηp2 = 0.093). In the comparison between the protocols, there was a significant difference between 60 and 90 s protocols (*p* = 0.045 *) of the multiple comparison (*p* = 0.047 *; ηp2 = 0.093; 95% CI = (−205.6400–1.4582).

**Figure 5:**
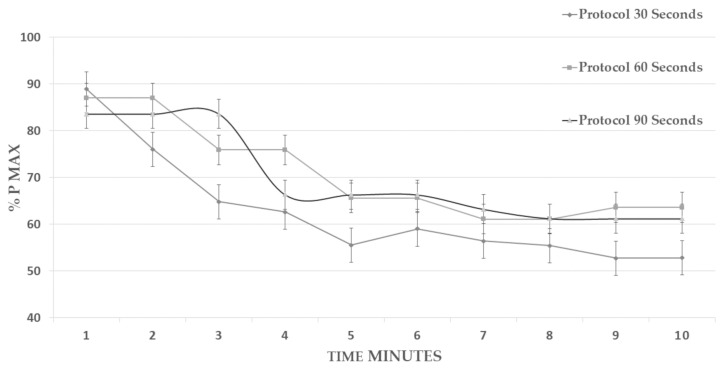
Development of maximum power of the different HIIT protocols.

Regarding Figure 5, the % of the maximum power developed, significant differences were found when comparing the three protocols (F = 7.991; *p* = 0.001; ηp2 = 0.202). Such comparison manifested a significant difference between the 30 and 90 s protocols (*p* = 0.001 *) of the multiple comparison (*p* = 0.001 *; ηp2 = 0.202; 95% CI = (0.0601–0.2417)).

## 4. Discussion

Due to the increasing popularity of HIIT, we wanted to analyze how protocols that vary in work/rest ratio and intensity influence physical responses, as they could be critical to effectively prescribing HIIT according to the training goal. In the BLa, there was no difference between the results at the end of the test in the three protocols (Table 2). Rønnestad [16] obtained results of a certain nature similar to ours, showing that two HIIT of different work/rest time, both short HIIT, 30 s of work and 15-s rest, and long HIIT, 5 min of work and 4-min rest with a goal of maximum effort, did not show differences regarding the production of BLa concentration at the end of the test. In line with these results, Tucker et al. [22], using two different protocols of 4-min intervals at 90–95% RH, separated by a 3-min recovery, and 16 1-min intervals at 90–95% of HR, separated by a recovery of 1 min, did not observe differences in the concentrations of Bla in blood at the end of the HIIT protocol (10.6 ± 1.5 vs. 10.6 ± 2.4 mmol-L); therefore, from 30 s of maximum intermittent effort, the Bla levels will possibly be similar regardless of the total work time.

Regarding HR, it has been observed that there were no differences between our protocols. Razanek et al. [23] also obtained similar results in the comparison between their protocols HR max (182.1 ± 6.2) and the same as Follador et al. [13] who compared 2 protocols of 10 intervals, one at 60 s/60 s HR max (150 ± 11.2) with another of 20 s/10 s with a HR (156.7 ± 11.9.) Furthermore, Patrick et al. [24] in their study obtained similar results regarding HR max between the HIIT protocols of 90 and 95% of HR max. HRmax has been shown to decrease in adaptation to resistance training [25]. In the study by Seiler and Hetlelid [26], 12 trained participants completed 6 and 4 min intervals with recovery intervals of different times (1 min, 2 min, and 4 min), reaching an average HRmax of 179 bpm for intervals of 1 and 2 min and 178 bpm for 4 min. These values are also similar to those obtained in the present study, possibly due to a greater similarity in the sample and the type of HIIT session developed. Regarding the results obtained in the CMJ jump test, no significant differences were observed pre-post-test, nor in the increase in height. This may be because the time between completion of the HIIT protocol and completion of the test may be long enough to restore potential loss of power in subjects. Other factors, in addition to lower extremity strength, affect vertical jump performance, with control of the ability to apply force (coordination) being a key element [27,28,29]. Regarding the powers during the session, the results found in the present investigation show that when the same total target intensity is used “all out”, the three protocols of 30 s, 60 s, and 90 s of work/rest interval produce quite similar results (Figure 5) In the 30 s protocol, between the first minute and the second minute, there is a loss of 70 w, but during the rest of the work, from minute 2 to 10, there are power losses of approximately 40 w (25%). In the 60 s protocol, from the start to the second minute, there is a 90 w power loss. Instead, from the second minute to the end of the test, there was a power loss of 50 w (24%). 

However, if we compare the mean power maintained (Figure 3) with the studies by Tucker et al. [22], where the mean power maintained was higher for the 16 × 1 (241 ± 45 W) compared to the 4 × 4 (204 ± 37 W), there was no significant difference, and Rønnestad et al. [16], with a power during the work intervals of 30 × 9 (363 ± 32 W) versus 4 × 5 (324 ± 42 W), also were without difference between their protocols. We observed differences in our study, though, which did obtain differences between the 30 s and 60 protocols and 30 s and 90 s, observing possible differences between a short HIIT and a long HIIT in terms of powers developed, although there is a greater predisposition to achieve greater power the shorter the work/rest interval. The 3 studies suggest the existence of differences in the nature of the intensity, and differences in work–rest time of the protocols, since Tucker et al. [22] worked at 90–95% HRmax, but Rønnestad et al. [16], and our study, worked at the maximum intensity that the subject could, although the HIIT work intervals throughout their study have a difference of more than 3 min of work break and 10 min of total time with ours. 

Olney et al. [30] have pointed out that responses to effort during HIIT may be influenced by the length of the interval. Kilpatrick et al. [31] compared perceived exertion responses in HIIT over 30 s, 60 s, and 120 s of maximum intensity work intervals and found that power loss increased from start to finish in all tests, with a greater effect during 120 s. The authors suggested that more frequent rest intervals between the shorter intervals would attenuate the increase in physical exertion, making it “easier” to maintain interval work over time. This is a positive finding because it can help fitness professionals and trainers with more options when prescribing HIIT.

## 5. Conclusions

The results of our research provide practical information for fitness professionals and trainers who prescribe HIIT. Firstly, the nature of the intensity type to be worked on will be a possible factor that can modify the test result, even when the rest of work/rest time variables are the same, and secondly, the HIIT of shorter work–rest time intervals allows one to maintain a higher power for a longer time, throughout the work session, without changes in the level of BLa or in the loss of jumping power.

## Figures and Tables

**Table 1:** Physiological control parameters chosen at the baseline for the 3 different training protocols.

Protocol High-Intensity Interval Training (HIIT)	30 s		60 s		90 s		
PMC	X	SD	X	SD	X	SD	Sig.
LA (mmol/L)	16.6	±0.6	15.9	±0.6	17.1	±0.60	0.373
HR_mean_ (b/min)	175.4	±8.4	170.7	±6.2	165.8	±6.06	<0.001

>HIIT = high intensity interval training, PMC = Physical measurements control, X = mean; SD = standard deviation; BLa = blood lactate concentration, HRmean = mean heart rate.

**Table 2:** Experimental physiological parameters chosen at the baseline for the 3 different training protocols.

Protocol HIIT	30 s		60 s		90 s		
PME	X	SD	X	SD	X	SD	Sig.
IA CMJ (cm)	−4.6	±3.3	−3.8	±3.6	−3.75	±3.6	0.682
IW CMJ (W/kg)	−2.9	±2.5	−2.8	±2.9	−2.37	±4.1	0.832
P_max_ (W)	531.7	±168.9	481.9	±139.2	355.6	±104.7	<0.001
P_mean_ (W)	312.6	±60.7	247.2	±54.1	223.4	±52.9	<0.001
P_loss_ (W)	−246.0	±188.5	−262.9	±145.3	−160.8	±68.8	0.047

HIIT = high intensity interval training, PME = Physical measurements experimental, X = mean; SD = standard deviation; IA CM J = increase height jump against movement, IW CM J = increased power jumping against movement, Pmax (W) = maximum power, Pmean (W) = mean power, Ploss (W) = loss power.

## Data Availability

The data presented in this study are available on request from the corresponding author.
